# A Retrospective Analysis of Urinary Tract Infections With Special Reference to the Phenotypic and Genotypic Detection of Fosfomycin-Resistant Escherichia coli Isolates at a Tertiary Care Center in South India

**DOI:** 10.7759/cureus.87359

**Published:** 2025-07-05

**Authors:** Vasuki M, Eunice Swarna Jacob, P Sivagamasundari, P B Praveen Kumar

**Affiliations:** 1 Department of Microbiology, Thanjavur Medical College, Thanjavur, IND

**Keywords:** escherichia coli, fosa3, fosc2, fosfomycin, urinary tract infections

## Abstract

One of the leading causes of morbidity worldwide is urinary tract infections (UTIs). We are already running out of antimicrobial drugs in this era of antimicrobial resistance. It is important to know the antimicrobial resistance pattern of all organisms that commonly cause human diseases.

The purpose of this investigation was to identify the pathogen that causes UTIs and to characterize the molecular makeup of the isolate of *Escherichia coli* that is resistant to fosfomycin in urine samples. A total of 1104 samples were examined during the June-August 2024 retrospective investigation at our institution. However, only 91 isolates of *Escherichia coli* were detected using standard microbiological methods. The Kirby-Bauer disk diffusion method was used to assess their susceptibility to antimicrobials. Of the 91 recovered *Escherichia coli*, 97% (n = 88) exhibited phenotypic susceptibility to fosfomycin, while the remaining 3 fosfomycin-resistant isolates underwent real-time polymerase chain reaction (RT-PCR). However, the fosA3 gene was detected in only one fosfomycin-resistant isolate.

While urinary tract infections are a common and widespread ailment that affects a large number of people worldwide, antibiotics are currently limited in their ability to treat them. Hence, fosfomycin may be the preferred choice of antibiotic.

## Introduction

The most prevalent infection that causes death worldwide is urinary tract infection. *Escherichia coli* is the most frequent bacterium responsible for urinary tract infections. Seventy to seventy-five percent of urinary tract infections are caused by the common bacterium *Escherichia coli* [1]. Acute uncomplicated urinary tract infections, uncomplicated pyelonephritis, and asymptomatic bacteriuria are the three categories of urinary tract infections. Healthy people can get an uncomplicated urinary tract infection, which is easily treated with antibiotics. Antimicrobial resistance and certain risk factors are frequently present in complicated urinary tract infections. Broad-spectrum antibiotics are used empirically to treat the majority of urinary tract infections. This results in multidrug resistance, which has grown to be a serious health issue [2].

The phosphonic acid derivative fosfomycin was initially identified in 1969. It stops the production of cell walls by turning off the enzyme uridine diphosphate-N-acetyl glucosamine enol pyruvyl transferase, which helps make peptidoglycans. The majority of bacterial resistance to fosfomycin is chromosomal in nature and is linked to mutations that disrupt the transport pathway for hexose phosphate absorption. Rarely, plasmid-mediated resistance is linked to the presence of the fosA gene, which codes for a protein that converts glutathione to fosfomycin, making it ineffective [3].

The purpose of this study was to identify the molecular characteristics of isolates of *Escherichia coli* that are resistant to fosfomycin in urine samples and to ascertain the prevalence of pathogens that cause urinary tract infections.

This article was partially presented as an oral presentation at the Chetimmunocon 2025 National Hybrid Conference on Recent Advances in Immunology on January 09, 2025.

## Materials and methods

Study design

This study was carried out at Thanjavur Medical College in Thanjavur, India, and was retrospective and cross-sectional.

Study place and population

The subjects of this study were hospitalized patients who were admitted between June 2024 and August 2024.

Sample collection

In this investigation, midstream clean catch urine samples were routinely collected from patients suspected of having a urinary tract infection in a sterile screw-capped container with a wide aperture.

Sample rejection criteria

Urine samples were summarily discarded with appropriate communication to the clinical team after being received in an inappropriate container or in a container that was either unlabeled or incorrectly labeled.

Sample processing

**Figure 1:**
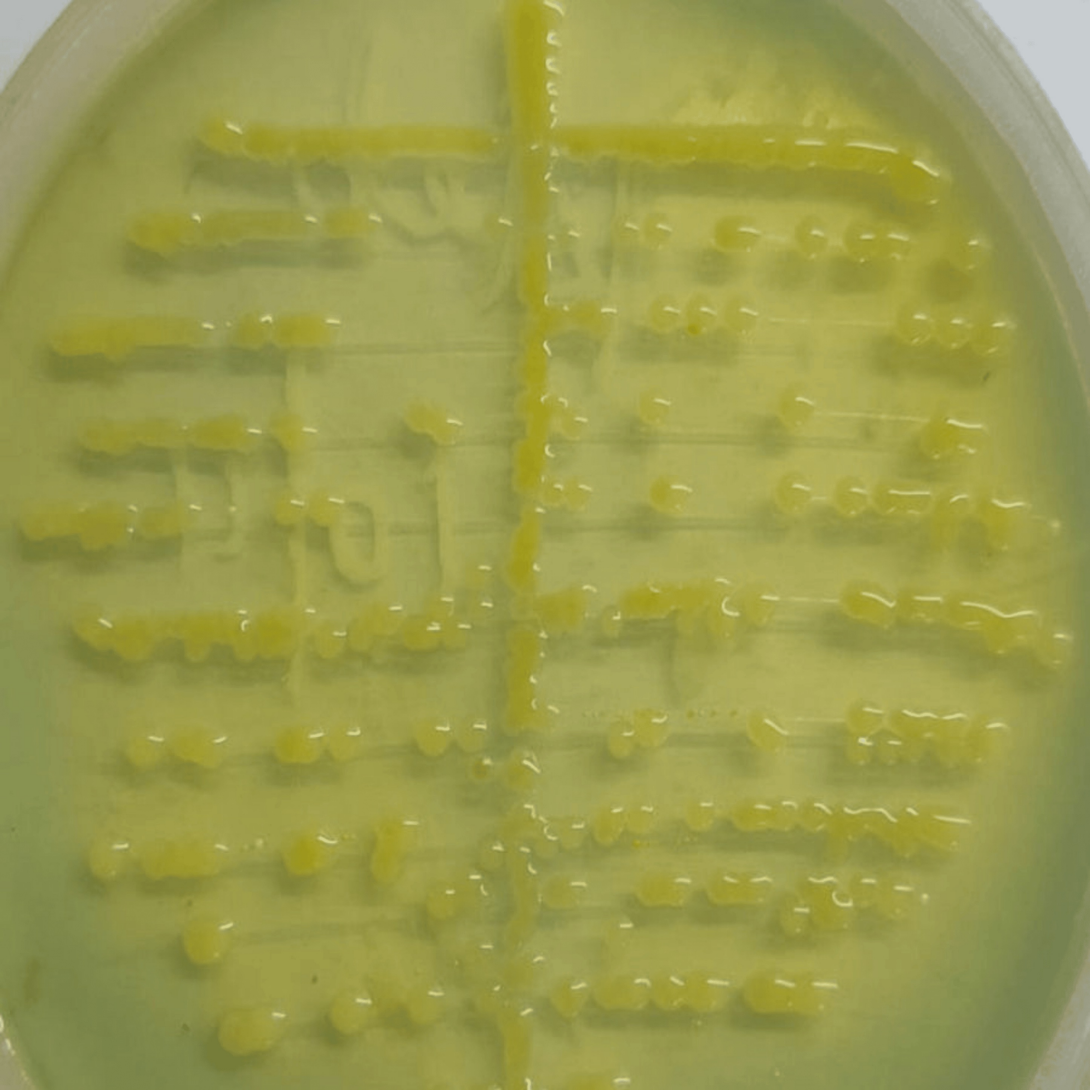
Semiquantitative streaking method in cysteine lactose electrolyte deficient (CLED) agar

**Figure 2:**
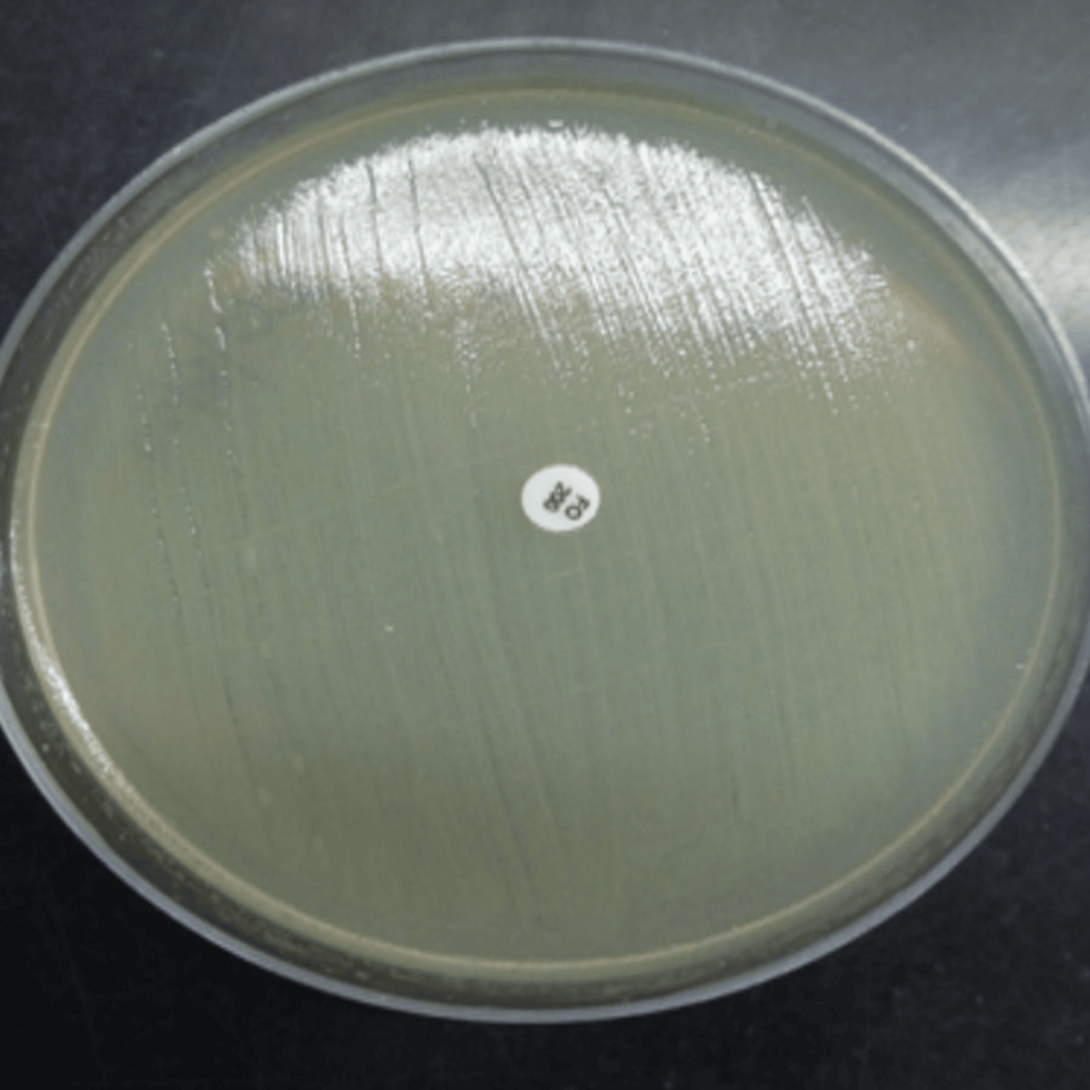
Kirby-Bauer disk diffusion method for antimicrobial susceptibility testing (AST)

All samples were subjected to a Gram stain and inoculated on cysteine lactose electrolyte deficient (CLED) agar using the semiquantitative streaking method (Figure 1). The plates were incubated aerobically at 37 degrees Celsius for 18 to 24 hours. After overnight incubation, colony morphology and characteristics were identified using conventional microbiological identification methods. According to Clinical Laboratory Standard Institute (CLSI) 2024 guidelines, antimicrobial susceptibility testing (AST) was done using the Kirby-Bauer disk diffusion method, and interpretation was made accordingly (Figure 2) [4].

Data analysis

The details of the culture-positive (n = 251) samples were collected retrospectively and entered into a Microsoft Excel sheet (Microsoft Corporation, Redmond, WA, US). Later, we prepared the antibiogram manually and analyzed the antimicrobial susceptibility pattern of each and every *Escherichia coli* isolate to know about the current status of all antimicrobials we tested in our laboratory.

## Results

**Figure 3:**
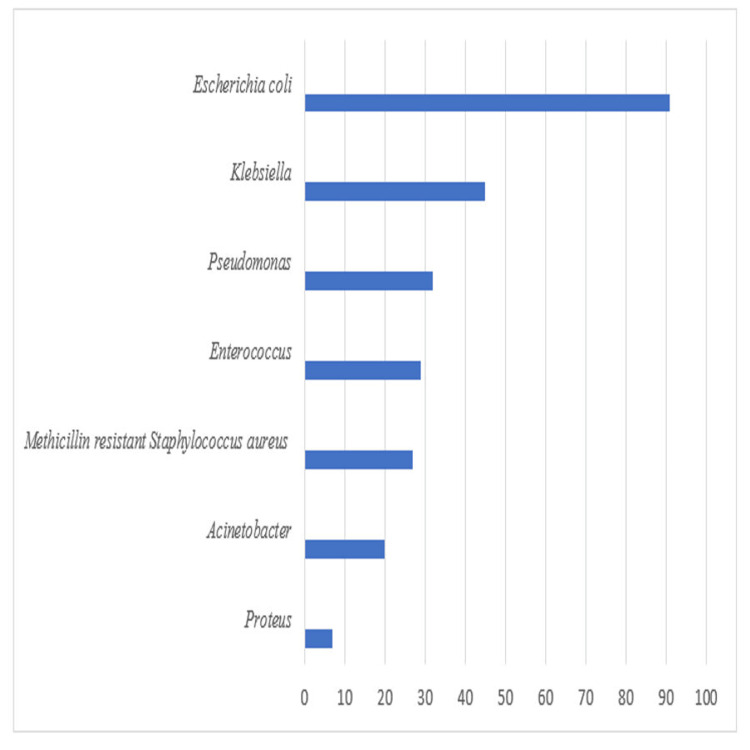
Organism-wise distribution of culture-positive urine isolates

Out of 1104 urine samples, 251 samples turned out to be positive (Figure 3). Among the 251 isolates, 91 isolates were found to be *Escherichia coli*, followed by *Klebsiella* (n=45), *Pseudomonas* (n=32), *Enterococcus* (n=29), methicillin-resistant *Staphylococcus aureus *(n=27), *Acinetobacter* (n=20), and *Proteus* (n=7).

**Figure 4:**
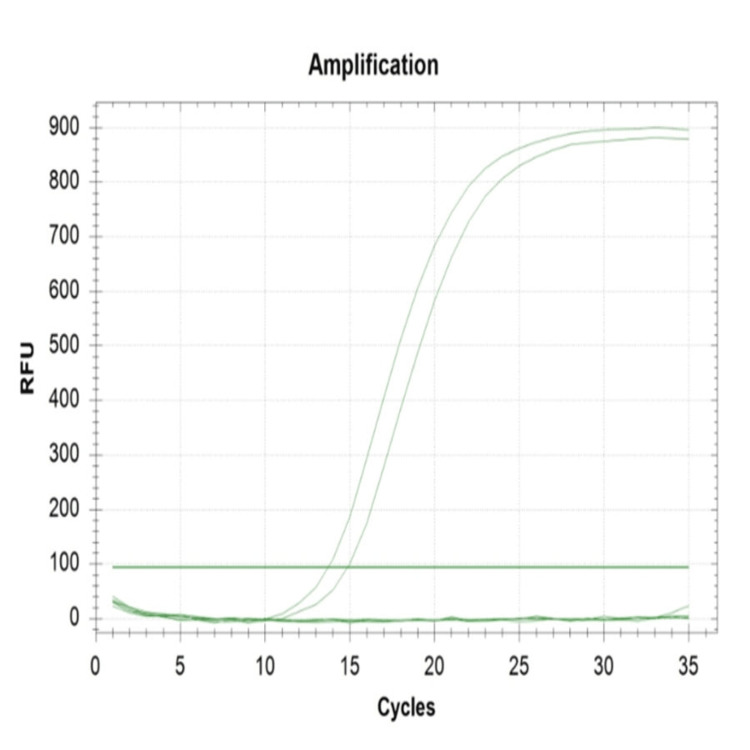
Real-time polymerase chain reaction of the fosA3 and fosC2 genes

The antimicrobial susceptibility pattern of all 91 *Escherichia coli* was documented in Table 1 based on the guidelines of the Clinical Laboratory Standards Institute (CLSI) 2024 [4]. Only three isolates were phenotypically shown to be resistant to fosfomycin and subjected to real-time polymerase chain reaction to detect the fosA3 and fosC2 genes; the fosA3 gene was detected in only one isolate (Figure 4).

**Table 1:** Antimicrobial susceptibility pattern of Escherichia coli isolates Clinical Laboratory Standards Institute (CLSI) 2024 tier definitions [4] Tier 1: Antimicrobial agents that are appropriate for routine, primary testing and reporting. Tier 2: Antimicrobial agents that are appropriate for routine, primary testing but may be reported following cascade reporting rules established at each institution. Tier 3: Antimicrobial agents that are appropriate for routine, primary testing in institutions that serve patients at high risk for MDROs but should only be reported following cascade reporting rules established at each institution. Tier 4: Antimicrobial agents that may warrant testing and reporting by clinician request if antimicrobial agents in other tiers are not optimal because of various factors. Urine Only: Antimicrobial agents designated by a “(U)” in tables should be reported only on organisms isolated from the urinary tract.

Antimicrobial agents	Susceptibility (in percentage)
Tier 1	
Ampicillin (n = 20)	21.9
Ceftriaxone (n = 39)	42.8
Gentamicin (n = 47)	51.6
Piperacillin tazobactam (n = 86)	94.5
Ciprofloxacin (n = 48)	52.7
Cotrimoxazole (n = 39)	42.8
Tier 2	
Amikacin (n = 45)	49.4
Meropenem (n = 86)	94.5
Tier 3	
Ceftazidime-avibactam (n = 65)	71.4
Tier 4	
Ceftazidime (n = 46)	50.5
Urine Only	
Nitrofurantoin (n = 38)	41.7
Fosfomycin (n = 88)	96.7

## Discussion

Urinary tract infections are a variety of infectious disorders that affect the urinary tract from the urethra to the kidneys. In females, it is more prevalent. Between 50% and 60% of women experience at least one episode over their lifetime [5]. Bacteria can enter the urinary tract in three different ways: ascending, hematogenous, and lymphatic. The ascending route is the most prevalent for acquiring infection [6]. The most frequent cause of uncomplicated UTIs is *Escherichia coli*, which is followed by *Klebsiella pneumoniae*, *Staphylococcus saprophyticus*, *Enterococcus faecalis*, Group B* streptococci*, and *Proteus mirabilis* [7]. A special phosphonic acid derivative, fosfomycin, is effective against a variety of Gram-positive and Gram-negative bacteria, such as *Enterococcus faecalis *and Enterobacterales, which are frequently responsible for uncomplicated urinary tract infections [8].

In our study, we found the most common urinary pathogen to be *Escherichia coli*, followed by *Klebsiella species*, the prevalence of the fosfomycin resistance rate was 3.2%, and the rate was significantly higher when compared with similar studies done by Yeliztanriverdi-Cayci et al., in which the fosfomycin resistance rate was 1.9% [9] and comparatively lower when compared with a study done in South Korea in which the fosfomycin resistance rate was 6.2% [10]. However, a study conducted in Uruguay indicated that the susceptibility rate to fosfomycin was 97.9%, which was a little higher than the susceptibility rate in our study [11]. Similarly, out of 1033 *Escherichia coli* isolates in research by Shakeel Mowlaboccus et al., only two isolates showed resistance to fosfomycin [12]. The most prevalent variant in Enterobacterales is fosA3, followed by fosA7 and fosA5 [13]. In our study, too, we found the fosA3 gene in an isolate.

Fosfomycin can be used as prophylactic therapy for patients with a risk of multidrug-resistant infection before any urological procedure. Even a single-dose regimen of fosfomycin can improve patient compliance with both the treatment and prevention of urinary tract infection since this drug is well excreted in the urine [14]. The growing interest in employing fosfomycin against multidrug-resistant (MDR) organisms in various indications led to the development of a parenteral formulation in the United States, first for complicated UTIs. It is unknown whether resistance will continue when other symptoms are incorporated, even though it has not been a problem in the case of an uncomplicated UTI [15].

Limitations and strengths

One of the main limitations of this study is its retrospective design, which may limit the completeness and consistency of data, and the study duration was only three months. Additionally, not all antibiotics listed in the Clinical Laboratory Standards Institute guidelines were tested [4], which may affect the comprehensiveness of the resistance profile. To the best of our knowledge, this study is the first to report fosfomycin resistance in *Escherichia coli* causing urinary tract infections in India, combining both phenotypic and genotypic detection methods.

## Conclusions

Globally, a large number of people suffer from urinary tract infections. Fosfomycin might be a better antibiotic in the current scenario, where treating urinary tract infections caused by multidrug-resistant strains of *Escherichia coli* can be challenging, and there are few antibiotic alternatives. *Escherichia coli* was the most prevalent isolate in this investigation. Three *Escherichia coli* isolates were found to be resistant to fosfomycin, although only one strain had the fosA3 gene. Nowadays, there is a tendency for fosfomycin resistance to rise with time. Therefore, further investigation and analysis are necessary to evaluate the potential fosfomycin resistance in the near future.
